# Meningiomas: A Comparative Study of ^68^Ga-DOTATOC, ^68^Ga-DOTANOC and ^68^Ga-DOTATATE for Molecular Imaging in Mice

**DOI:** 10.1371/journal.pone.0111624

**Published:** 2014-11-04

**Authors:** María Luisa Soto-Montenegro, Santiago Peña-Zalbidea, Jose María Mateos-Pérez, Marta Oteo, Eduardo Romero, Miguel Ángel Morcillo, Manuel Desco

**Affiliations:** 1 Unidad de Medicina y Cirugía Experimental, Instituto de Investigación Sanitaria Gregorio Marañón, Madrid, Spain; 2 Centro de Investigación Biomédica en Red de Salud Mental (CIBERSAM), Madrid, Spain; 3 Departamento de Bioingeniería e Ingeniería Aerospacial, Universidad Carlos III, Madrid, Spain; 4 Unidad de Aplicaciones Biomédicas y Farmacocinética, Centro de Investigaciones Energéticas, Medioambientales y Tecnológicas (CIEMAT), Madrid, Spain; Genentech, United States of America

## Abstract

**Purpose:**

The goal of this study was to compare the tumor uptake kinetics and diagnostic value of three ^68^Ga-DOTA-labeled somatostatin analogues (^68^Ga-DOTATOC, ^68^Ga-DOTANOC, and ^68^Ga-DOTATATE) using PET/CT in a murine model with subcutaneous meningioma xenografts.

**Methods:**

The experiment was performed with 16 male NUDE NU/NU mice bearing xenografts of a human meningioma cell line (CH-157MN). ^68^Ga-DOTATOC, ^68^Ga-DOTANOC, and ^68^Ga-DOTATATE were produced in a FASTLab automated platform. Imaging was performed on an Argus small-animal PET/CT scanner. The SUV_max_ of the liver and muscle, and the tumor-to-liver (T/L) and tumor-to-muscle (T/M) SUV ratios were computed. Kinetic analysis was performed using Logan graphical analysis for a two-tissue reversible compartmental model, and the volume of distribution (V_t_) was determined.

**Results:**

Hepatic SUV_max_ and V_t_ were significantly higher with ^68^Ga-DOTANOC than with ^68^Ga-DOTATOC and ^68^Ga-DOTATATE. No significant differences between tracers were found for SUV_max_ in tumor or muscle. No differences were found in the T/L SUV ratio between ^68^Ga-DOTATATE and ^68^Ga-DOTATOC, both of which had a higher fraction than ^68^Ga-DOTANOC. The T/M SUV ratio was significantly higher with ^68^Ga-DOTATATE than with ^68^Ga-DOTATOC and ^68^Ga-DOTANOC. The V_t_ for tumor was higher with ^68^Ga-DOTATATE than with ^68^Ga-DOTANOC and relatively similar to that of ^68^Ga-DOTATOC.

**Conclusions:**

This study demonstrates, for the first time, the ability of the three radiolabeled somatostatin analogues tested to image a human meningioma cell line. Although V_t_ was relatively similar with ^68^Ga-DOTATATE and ^68^Ga-DOTATOC, uptake was higher with ^68^Ga-DOTATATE in the tumor than with ^68^Ga-DOTANOC and ^68^Ga-DOTATOC, suggesting a higher diagnostic value of ^68^Ga-DOTATATE for detecting meningiomas.

## Introduction

Meningiomas arise from the meningothelial cells of the arachnoid membranes, which are attached to the inner layer of the dura mater [1]. With a yearly incidence of approximately 7.44/100,000, they account for 35% of primary intracranial tumors [2]. Meningiomas are usually diagnosed using morphologic imaging methods such as computed tomography (CT) and magnetic resonance imaging (MRI). However, meningiomas located near the base of the skull may be difficult to distinguish from other lesions, such as lymphomas, metastases, or neurinomas. Consequently, management of meningiomas at these sites requires a specific therapeutic approach [3]. Functional imaging techniques could be advantageous for detecting meningiomas in cases where biopsy is risky (eg, location near critical intracranial structures) and for tumors located at the skull base, with possible infiltration of bone structures.

Meningiomas express a large variety of receptors, including progesterone, androgens, growth factor, prolactin, dopamine, and somatostatin receptor subtype 2 (SSTR2) [4], [5]. Abundant expression of SSTRs is a characteristic of many types of tumors, mainly neuroendocrine tumors (NETs), lung cancer, lymphomas, and meningiomas. To date, five different SSTR subtypes have been identified (SSTR1–5). Meningiomas express relatively high levels of SSTR2, thus making them ideal targets for functional imaging and radionuclide therapy with radiolabeled somatostatin analogues [5], [6]. These receptors can be visualized *in vivo* by targeted positron emission tomography (PET) tracers.

^68^Ga-DOTA–labeled somatostatin analogues are PET tracers that bind specifically to somatostatin receptors (SSTRs). ^68^Ga has clear advantages: it has a short half-life (68 minutes), which facilitates its application in clinical practice, and can be produced with a ^68^Ge/^68^Ga radionuclide generator. The 3 compounds most widely used in PET functional imaging are ^68^Ga-DOTATOC (^68^Ga-DOTA -Tyr^3^-octreotide), ^68^Ga-DOTANOC (^68^Ga-DOTA-Nal^3^-octreotide), and ^68^Ga-DOTATATE (^68^Ga-DOTA-Tyr^3^-octreotate). ^68^Ga-DOTATOC and ^68^Ga-DOTATATE are commonly used for PET/CT imaging of SSTRs. Their high affinity has been demonstrated for SSTR2, which is one of the most common SSTR subtypes found in tumors [7]. ^68^Ga-DOTANOC targets a broader range of somatostatin subtype receptors, including SSTR2, SSTR3, and SSTR5 [8]. Preliminary results in humans suggest that this new radiopeptide identifies more metastases than SSTR2-specific tracers [9]. However, it is not yet clear which of these somatostatin analogues provides better results in the case of meningiomas.

^68^Ga-DOTATOC is the most commonly used radiotracer for imaging meningioma [10], [11], [12]. The ability of ^68^Ga-DOTATOC to adequately detect this tumor has proven useful for planning radiation therapy. Moreover, ^68^Ga-DOTATOC-PET data can complement anatomical data from MRI and CT to improve target volume definition, especially in cases with complex infiltration and recurrent disease after surgery [13], [14]. In fact, recent progress in the development of PET radiotracers has enabled PET/CT imaging of various SSTRs, and ^68^Ga-DOTA–labeled somatostatin analogues have been reported to show higher sensitivity for the detection of NETs and other types of tumors than the most widely used radiotracer, 2-deoxy-2-[^18^F] fluoro-D-glucose, which measures glucose metabolism [15], [16].

To our knowledge, the PET radiotracers ^68^Ga-DOTATOC, ^68^Ga-DOTANOC, and ^68^Ga-DOTATATE have not been directly compared in terms of tumor uptake and ability to detect meningiomas. Therefore, the goal of this study was to compare the tumor uptake kinetics and diagnostic value of these three ^68^Ga-DOTA-labeled somatostatin analogues in a PET/CT animal model with subcutaneous human meningioma xenografts.

## Materials and Methods

### 1. Cell culture

CH-157MN tumor cells were provided by Randy Jensen, from the Department of Neurosurgery of the University of Utah Health Care. This human malignant meningioma cell line exhibits microscopic, immunohistochemical, and ultrastructural features of meningioma [17], [18]. Cells were grown in Dulbecco’s Modified Eagle’s Medium (DMEM) (from Sigma-Aldrich) containing 10% fetal bovine serum (FBS), 2 mM L-glutamine, 50 units/ml penicillin, and 50 µg/ml streptomycin. Cultures were maintained at 37°C and in 5% CO_2_. Cell viability was over 90%, as determined by trypan-blue staining.

### 2. Meningioma mouse flank xenograft model

The experiment was performed with 16 male NUDE NU/NU mice. The animals were purchased from Charles River Laboratories (Spain), maintained at a constant temperature (24±0.5°C) under a 12 hour light/dark cycle, and permitted free access to commercial rodent laboratory chow and water. All experimental procedures were conducted in conformity with European Communities Council Directive 2010/63/EU and approved by the Ethics Committee for Animal Experimentation of our hospital (Comité de Ética de Experimentación Animal, CEEA; number ES280790000087).

A CH-157MN tumor cell suspension containing 1.5×10^6^ cells in a volume of 0.1 ml was injected subcutaneously into both flanks using a 30-gauge needle to increase the probability of growth.

The tumor size threshold to start PET/CT imaging was 0.1 cc.

### 3. Synthesis of ^68^Ga-DOTA-peptides

The precursors were obtained from BCN Peptides (Barcelona, Spain) (DOTATOC) and ABX GmbH (Radeberg, Germany) (DOTATATE and DOTANOC). The reagents HCl 30%, ethanol, acetonitrile, and HEPES were obtained from Merck; NaCl was obtained from Sigma Aldrich.

Most of the synthesis was performed using an automated process programmed in a FastLab module (General Electric Healthcare). Before synthesis was started, the precursor (11.37, 11.13, and 10.98 nmols of DOTATOC, DOTATATE, and DOTANOC, respectively) and HEPES were introduced into the needle of the reactor. ^68^Ga was obtained from a 50-mCi Obninsk ^68^Ge/^68^Ga radionuclide generator (Eckert & Ziegler) by elution with HCl 0.1N.

In this step, we applied a fractionation method, keeping only the middle (most active) part of the eluate. The effluent containing the ^68^Ga was stored in a reservoir, and HCl 30% was added. The mixture was passed through a SAX cartridge (SPE-Spezialkartuschen Chromafix PS-HCO3, Macherey-Nagel) for purification and concentration of ^68^Ga, which was eluted from the cartridge to the reactor using 200 µL of ultrapure water. The mixture was heated at 95°C for 10 minutes for labeling. Once the reaction had finished, the reactor was washed with ultrapure water to obtain the ^68^Ga-DOTA-peptide, which was purified by passing it through a C18 cartridge (Sep-Pak SPE Waters). The product was eluted from the Sep-Pak with ethanol and heated at 120°C for evaporation of ethanol and sterilization. The radiotracer was finally formulated in phosphate-buffered saline (PBS)/NaCl (1/3).

The protocol used for the synthesis of ^68^Ga-DOTATATE and ^68^Ga-DOTANOC was slightly different from that of ^68^Ga-DOTATOC. Changes included a second elution of the anion exchange cartridge that duplicated the volume in the reactor, thus increasing acidity (pH = 4.5 for ^68^Ga-DOTATOC and pH = 4.0 for ^68^Ga-DOTATATE and ^68^Ga-DOTANOC). Also, the reactor was washed twice to recover more activity. The reason for these changes was to improve the radiochemical yield that increased from 37.8% to 67.8% for ^68^Ga-DOTATATE and from 43% to 66.1% for ^68^Ga-DOTANOC.

Quality controls were carried out before administration of the tracer. The products were clear and colorless. pH was measured using pH paper strips, which consistently yielded values of around 7. The purity of the radiochemical was measured in a 1200 Agilent HPLC system, the average of all tracers being greater than 98%. Radiosynthesis values are expressed as mean ± SEM (standard error of mean).

### 4. Determination of SSTR affinity

Affinity was determined from three independent experiments performed in triplicate and was measured by means of a homologous competitive binding experiment according to Motulsky and Neubig [19]. The IC_50_ (concentration of cold ligand [somatostatin-28] that reduces specific binding of radioligand by 50%) was determined by competitive binding assays with [^125^I]-somatostatin-28 (Amersham Pharmacia Biotech, UK) in CH-157MN meningioma cells. Briefly, cells were incubated into a 24-well cell culture plate (2–2.5×10^5^ cells/well) at 37°C for 1 hour in a CO_2_ incubator with 0.5 nM [^125^I]-somatostatin-28 (Amersham Pharmacia Biotech, UK) in the presence of increasing concentrations of somatostatin-28 (0.1 nM-10 µM) in 0.25 ml of binding media (DMEM, 2 mM L-glutamine, 50 units/ml penicillin and 50 µg/ml streptomycin). The reaction medium was aspirated after incubation. Cells were rinsed with 0.5 ml of ice-cold PBS twice and lysed in 0.5 ml of 1 N NaOH for five minutes. The activity in cells was measured in a Packard Cobra II gamma counter. The competitive binding curves were obtained by plotting the [^125^I]-somatostatin-28 bound to cells against concentrations of cold ligand. IC_50_ and maximum numbers of binding sites (*B_max_*) were calculated using Origin 7.5 software (OriginLab Corporation, Northampton, Massachusetts, USA).

### 5. PET imaging studies

**Figure 1:**
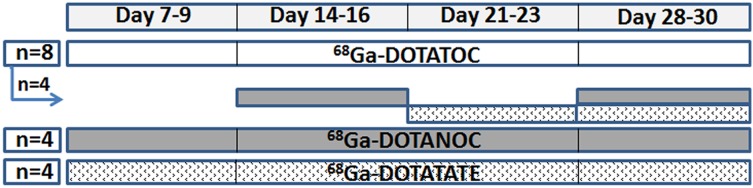
Experimental protocol. Animals were imaged with ^68^Ga-DOTATOC (white line), ^68^Ga-DOTANOC (gray line) and/or ^68^Ga-DOTATATE (dotted line). Half of the animals imaged with ^68^Ga-DOTATOC were also imaged with the other two radiotracers in alternate days with respect to ^68^Ga-DOTATOC (n represents the number of animals).

Animals were scanned on a small-animal PET scanner (ARGUS PET-CT, SEDECAL, Madrid, Spain) under anesthesia (isoflurane, 3% induction and 1.5% maintenance in 100% O_2_). Imaging was performed 7–28 days after tumor cell inoculation. Animals were imaged over four consecutive weeks (at days 7, 14, 21 & 28 after cell inoculation) with one radiotracer (n = 8 with ^68^Ga-DOTATOC; n = 4 with ^68^Ga-DOTANOC and n = 4 with ^68^Ga-DOTATATE). Half of the animals imaged with ^68^Ga-DOTATOC were also imaged with the other two radiotracers in alternate days with respect to ^68^Ga-DOTATOC (Figure 1). Overall, n values for the PET study were: 27 for ^68^Ga-DOTATOC, 20 for ^68^Ga-DOTANOC and 21 for ^68^Ga-DOTATATE.

The tracers (^68^Ga-DOTATOC, ^68^Ga-DOTANOC, or ^68^Ga-DOTATATE) were injected into the tail vein (mean, 11.47 MBq; range, 9.25–20.35 MBq) and a dynamic study centered in the tumor was acquired for 90.3 minutes (112 frames: 20×10 s, 10×30 s, and 82×60 s). Frames corresponding to the last 60 minutes of the study were summed to form a static image. Images were reconstructed using the 2D–OSEM (Ordered Subset Expectation Maximization) algorithm (16 subsets and two iterations), which yields a spatial resolution for this scanner of 1.45 mm Full Width at Half Maximum (FWHM), with a voxel size of 0.3875×0.3875×0.7750 mm^3^. The energy window was 400–700 keV. Decay and deadtime corrections were applied.

### 6. CT imaging study

CT images were acquired using the PET/CT scanner with the following parameters: 320 mA, 45 KV, 360 projections, eight shots, resulting in 200 µm of resolution. CT images were reconstructed using a Feldkamp algorithm after obtaining an isotropic voxel size of 0.125 mm [20]. Thanks to the intrinsic alignment of the PET/CT device, these anatomical images did not require any registration with their corresponding PET scans and were used to draw the various regions of interest (ROIs) used in this study.

### 7. Analysis of PET-CT data

Three 3D ROIs were drawn manually on the CT image, one for each tissue of interest (tumor, liver, and muscle), plus a background ROI outside the body area. Since the CT images are intrinsically registered to the PET images, the ROIs drawn on the CT image were directly overlaid on the PET image after correction for the different image sizes. The assessment of PET data included calculation of the maximum standard uptake value (SUV_max_) on the static tomographic study and computation of the volume of distribution (V_t_) from the dynamic study.

#### a) Static study

Tumor, muscle, and liver SUV_max_ values at 30–90 minutes post-injection were recorded. As our field of view did not include the brain, the usual localization of this type of tumor, we selected two reference tissues: the liver, which consistently shows a slight to moderate physiologic SSTR density, and muscle, with low SSTR expression, similar to that of the brain [21]. The SUV_max_ of the tumor lesions was normalized to the SUV_max_ of the liver (T/L) and muscle (T/M) [21]. The T/L and T/M SUV ratios were considered markers of the ability of the radiopharmaceutical to localize in the tumor.

#### b) Dynamic study

V_t_ was calculated from dynamic PET data using standard Logan graphical analysis for a two-tissue reversible compartmental model [22]. Image-derived input functions and tissue time-activity curves were obtained by manually drawing ROIs over the static PET image (artery) or CT image (liver, muscle, and tumor). The artery used was the descending aorta, because the spillover from other organs is low and the descending aorta extends from the upper chest to the lower abdomen [23]. The model was resolved using the tracer kinetic modeling library [24].

### 8. Immunohistochemistry

The animals were sacrificed in a CO_2_ chamber. Tumors were extracted and fixed in 10% formalin solution. Five-micrometer paraffin sections were used for immunohistochemical analysis. High temperature antigen unmasking (microwaving of slides in 0.01-M citrate buffer for 10 minutes) was used after deparaffinization to enhance staining. Sections were incubated with 5% horse serum for 30 minutes to block the Fc receptor in tissue and then washed three times with sterile PBS (pH 7.5) before incubation with polyclonal antibody IgG anti-SSTR2 (Sigma-Aldrich, St. Louis, Missouri, USA) and dilution (1/50) in PBS/bovine serum albumin [25]. Goat anti-rabbit IgG-peroxidase secondary antibody was purchased from Sigma-Aldrich (St. Louis, Missouri, USA). For immunohistochemistry, peroxidase was visualized using diaminobenzidine as a substrate (Vector Laboratories, Burlingame, CA, USA). The sections were counterstained with hematoxylin.

### 9. Statistical analysis

The data analysis only included tumors grown in the left flank. SUV_max_ and V_t_ are presented as mean ± standard error of mean (SEM). Homogeneity of variance was assessed using Levene’s test. Data were analyzed using one-way analysis of variance (ANOVA) followed by post hoc tests (Tukey’s HSD) when statistical significance was reached (p<0.05).

## Results

### Radiosynthesis

Radiosynthesis was successfully performed with the three somatostatin analogues. For ^68^Ga-DOTATOC, ^68^Ga-DOTANOC and ^68^Ga-DOTATATE respectively, the mean activity (MBq) was 232.73±14.06, 275.28±14.80 and 274.90±10.36; the decay-corrected radiochemical yield (%) was 54.03±2.45, 66.13±3.35 and 67.81±3.12; the radiochemical purity (%) was 97.86±0.51, 99.38±0.31 and 99.68±0.13 and the injected specific activity (MBq/nmol) was 11.07±1.09, 12.45±1.29 and 12.33±1.32.

### Tumor growth

All animals developed tumors on both flanks, although only left side tumors were included in the data analysis. Tumor volumes were 0.13±0.01 cc (7–9 days), 0.73±0.10 cc (14–16 days), 1.55±0.21 cc (21–23 days) and 1.55±0.32 cc (28–30 days). Tumor growth showed a second grade polynomial trend with an R^2^ = 0.9619.

### *In vitro* binding study

**Figure 2:**
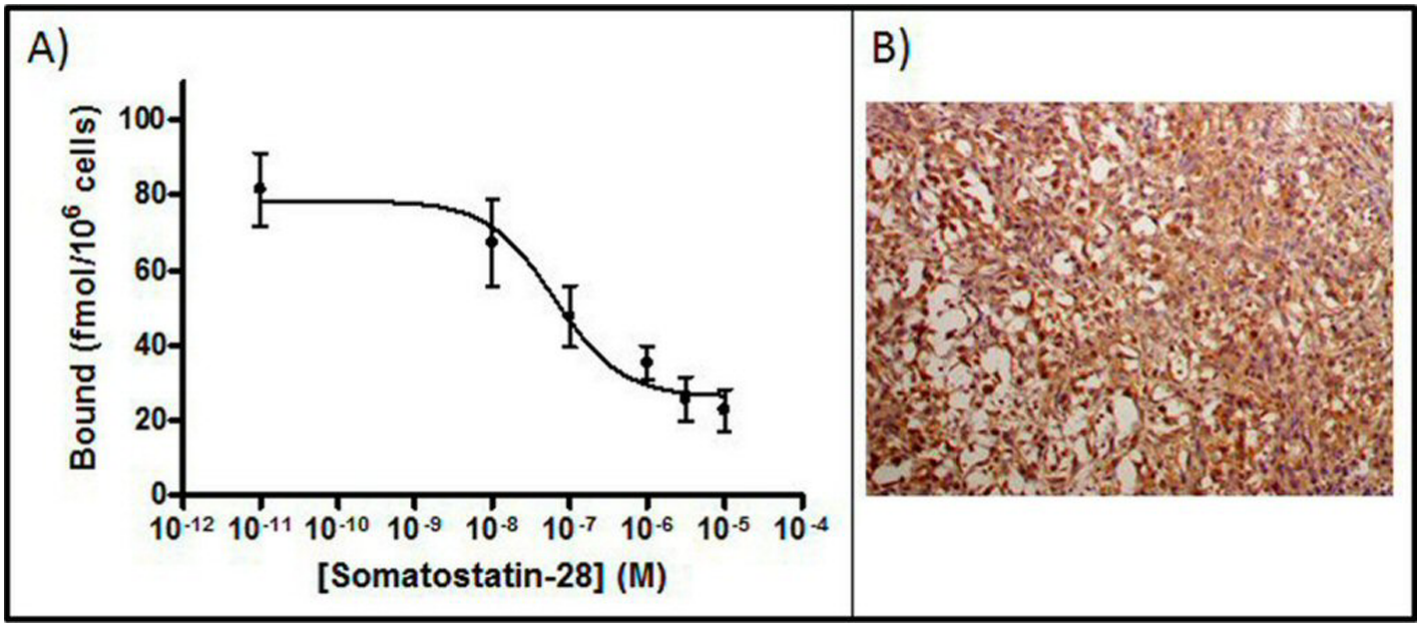
SSTR affinity and immunohistochemistry studies. A) Competitive binding of [^125^I]-Somatostatin-28 to CH-57MN cells incubated at 37°C. IC_50_ and maximum numbers of binding sites (*B_max_*) were calculated using Origin 7.5 software. Data are from three independent experiments performed in triplicate (Mean ± SEM). B) Immunohistochemistry using SSTR2 antibody (original magnification, x20). Tumor obtained from the CH-157MN cell line.

Figure 2A shows the high binding affinity of SSTR in CH-157MN cells for the universal ligand somatostatin-28. The IC_50_ was 63.7±32.3 nM and the B_max_ was 78.4±6.5 fmol/10^6^ cells.

### Immunohistochemistry

Immunohistochemical analysis showed that the tumor expressed SSTR2 (Figure 2B); staining was more intense on the periphery of the cells and showed weaker reactivity inside them.

### Static imaging study: SUV_max_ analysis

**Figure 3:**
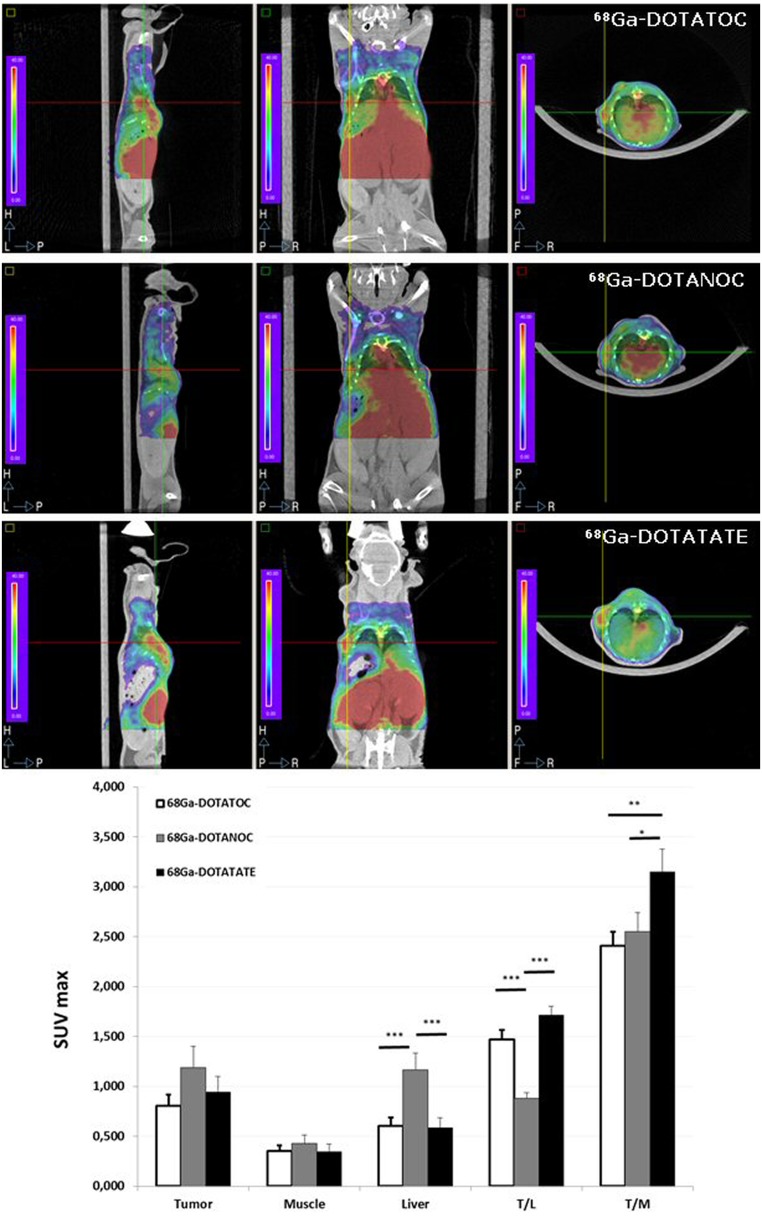
Tumor uptake visualized by PET. From top to bottom (^68^Ga-DOTATOC, ^68^Ga-DOTANOC, and ^68^Ga-DOTATATE), PET/CT images show radiotracer uptake in the tumor of the same animal. The graph shows the mean SUV_max_ for muscle, liver, T/L, and T/M ratios for each radiotracer. Values are expressed as mean ± SEM (***p<0.001, ^68^Ga-DOTATOC and ^68^Ga-DOTANOC; ^$$$^p<0.001, ^68^Ga-DOTATOC and ^68^Ga-DOTATATE; ^&&&^p<0.001, ^68^Ga-DOTANOC and ^68^Ga-DOTATATE) (n* = *20–27 images per group).

Figure 3 shows the mean SUV_max_ for tumor, muscle, and liver, as well as the T/L and T/M SUV ratios for each radiotracer. The ANOVA revealed significant differences in liver uptake between radiotracers (p<0.001, F = 9.164), with ^68^Ga-DOTANOC showing higher uptake than ^68^Ga-DOTATOC or ^68^Ga-DOTATATE (p<0.001). No significant differences between tracers were found for SUV_max_ in tumor and muscle tissue.

The ANOVA also revealed significant differences in T/L SUV ratio between radiotracers (p<0.001, F = 19.646), with ^68^Ga-DOTANOC showing lower uptake than ^68^Ga-DOTATOC and ^68^Ga-DOTATATE (p<0.001). No differences were found between ^68^Ga-DOTATATE and ^68^Ga-DOTATOC.

As for differences in the T/M SUV ratio, ANOVA revealed significant differences between the radiotracers (p<0.01, F = 5.560), with ^68^Ga-DOTATATE uptake proving to be higher than that of ^68^Ga-DOTATOC (p<0.01) and ^68^Ga-DOTANOC (p<0.05).

Figure 3 shows comparative PET/CT images of the same animal for the three radiotracers. The tumors in the flank were better visualized with 68Ga-DOTATOC and 68Ga-DOTATATE than with 68Ga-DOTANOC.

### Dynamic study: V_t_ analysis

ANOVA revealed significant differences in V_t_ between radiotracers in the tumor (p = 0.0132, F = 5.349) and radiotracers in the liver (p = 0.0013, F = 10.706). Post hoc tests revealed significant differences between ^68^Ga-DOTANOC and ^68^Ga-DOTATOC (p<0.01 for tumor and p<0.05 for liver). Statistically significant differences were also found between ^68^Ga-DOTANOC and ^68^Ga-DOTATATE (p<0.05 for tumor and p<0.05 for liver).

**Figure 4:**
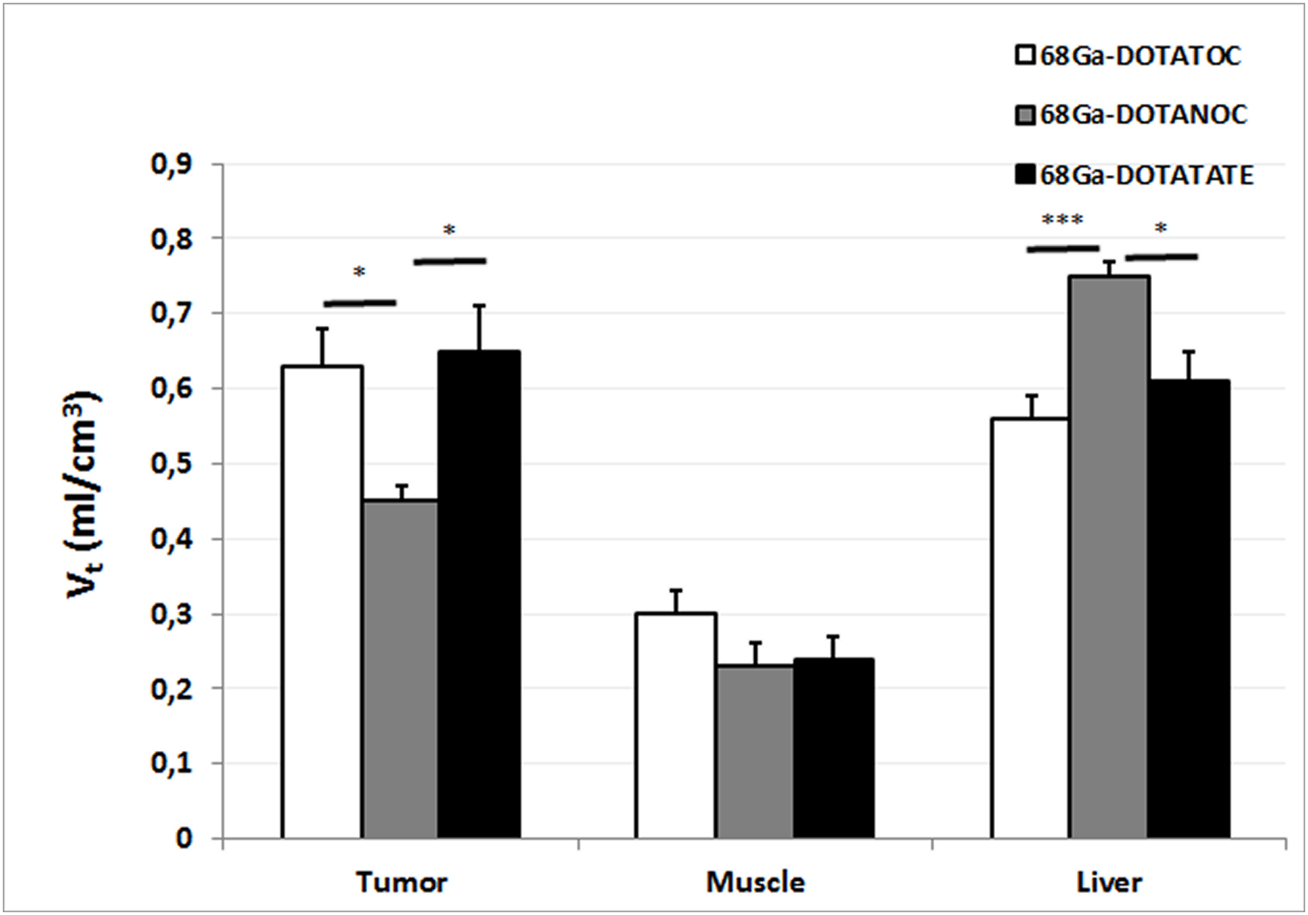
Plots of volume of distribution (V_t_) (ml/cm^3^) for liver, muscle and tumor for each radiotracer. Values are expressed as mean ± SEM (*p<0.05; **p<0.01; ***p<0.001) (n* = *7–10 images per group).

No significant differences were found for V_t_ in muscle. Figure 4 shows the V_t_ (liver, muscle, and tumor) for each radiotracer.

## Discussion

To our knowledge, this is the first report to compare the tumor uptake and diagnostic value of the three ^68^Ga-DOTA-labeled somatostatin analogues ^68^Ga-DOTATOC, ^68^Ga-DOTANOC, and ^68^Ga-DOTATATE using PET/CT in an animal model with a subcutaneous meningioma xenograft. Our study demonstrates the ability of the three radiolabeled somatostatin analogues to image mice bearing human meningioma xenografts.

Only three papers have evaluated the kinetics of ^68^Ga-DOTA-labeled somatostatin analogues by PET in humans. The first focused on meningiomas located near the skull base [26]. The kinetics of ^68^Ga-DOTATOC was analyzed using a two-tissue compartmental model, which revealed significant differences between meningiomas and the reference tissue, with high values in the tumor for vascular fraction, receptor binding, and K_1_/k_2_ and k_3_/k_4_ ratios. These data highlight that the main mechanisms responsible for tracer accumulation are receptor binding and trapping by internalization. The second and third studies compared the kinetics of ^68^Ga-DOTATOC and ^68^Ga-DOTATATE in NETs [27], [28] and did not reveal significant differences in kinetic behavior between the tracers. Our results are consistent with those of these studies: no significant differences in V_t_ were found between the two tracers, suggesting that the human meningioma cell line used had predominantly SSTR2, since both radiotracers exhibit mostly SSTR2-selective binding. Moreover, our binding and immunohistochemical studies showed that the CH-157MN meningioma cells expressed SSTR2 and, consequently, suggest that both radiotracers could be equally used for imaging and staging patients with meningioma. On the contrary, V_t_ in the tumor was lower for ^68^Ga-DOTANOC than for the other two tracers, and although ^68^Ga-DOTANOC has higher affinity for SSTR2 than ^68^Ga-DOTATOC, it also has affinity for SSTR3 and SSTR5 [29]. Consequently, there seems to be no advantage to using a tracer that targets a broader range of somatostatin subtype receptors when imaging meningioma.

We recorded significant differences between radiotracers in the SUV of the liver and the T/L and T/M SUV ratios. Uptake of ^68^Ga-DOTANOC in the liver was greater than that of the other tracers, probably because ^68^Ga-DOTANOC is more lipophilic than ^68^Ga-DOTATATE and ^68^Ga-DOTATOC, resulting in increased accumulation of tracer in the liver [8]. The T/L SUV ratio was similar for both ^68^Ga-DOTATOC and ^68^Ga-DOTATATE and higher than that of ^68^Ga-DOTANOC, presumably because of the lower uptake of these tracers than of ^68^Ga-DOTANOC in the liver. This finding must be taken into account if the tumor is located in the liver. On the other hand, the T/M SUV ratio for ^68^Ga-DOTATATE was significantly higher than that obtained with ^68^Ga-DOTATOC or ^68^Ga-DOTANOC. Normalization to a tissue with moderate SSTR expression (liver) or to a tissue with low SSTR expression (muscle) yields slightly different results, indicating that the organ distribution of these somatostatin analogues differs depending on the peptide used in the radiotracer. Since expression of SSTR is similar in both muscle and brain tissue, muscle was the tissue chosen for normalization in our study. In this respect, uptake of ^68^Ga-DOTATATE in the tumor was greater than that of the other two tracers, suggesting that ^68^Ga-DOTATATE might be considered the radiotracer of choice for detection of meningioma in the brain.

Few authors have compared the SUV_max_ of [^68^Ga]-labeled somatostatin analogues, and none were assessed in meningiomas. Poeppel et al. showed that the SUV_max_ of ^68^Ga-DOTATOC tended to be higher than that of ^68^Ga-DOTATATE [7], [21]. Velikyan et al. found no statistically significant differences in tumor uptake between ^68^Ga-DOTATOC and ^68^Ga-DOTATATE, but suggested that the slight difference in healthy organ distribution may render ^68^Ga-DOTATATE preferable for planning of peptide receptor radionuclide therapy [28], [30]. Yang et al. suggested that ^68^Ga-DOTATATE may be more sensitive and specific than ^68^Ga-DOTATOC [31]. Our results are in line with those of previous works with NETs, and the organ distribution for ^68^Ga-DOTATATE and ^68^Ga-DOTATOC is also very similar. Only two studies have compared tumor detection rates of ^68^Ga-DOTATATE and ^68^Ga-DOTANOC in patients with NETs [32], [33]. Wild et al. detected significantly more lesions with ^68^Ga-DOTANOC than with ^68^Ga-DOTATATE [33], probably owing to its broader somatostatin receptor binding profile [29]. On the contrary, Kabasakal et al. demonstrated that images obtained with ^68^Ga-DOTATATE and ^68^Ga-DOTANOC could have comparable diagnostic accuracy for NETs, although ^68^Ga-DOTATATE showed a higher uptake and may have a potential advantage over ^68^Ga-DOTANOC [32]. Our results agree with those of Kabasakal et al. and provide further evidence in favor of ^68^Ga-DOTATATE as the first-choice radiotracer or of ^68^Ga-DOTATOC as the second choice in the case of meningiomas.

Our study has several limitations. The first is the low anatomical resolution of the PET images. Measurements of metabolic activity by this technique in small regions may not be entirely accurate, since the activity of the ROI could be contaminated by that of surrounding regions. However, partial volume effects were minimized by defining the ROI on a registered CT scan of the same animal. Second, although partial volume effects were not corrected in the kinetic analysis, we were not measuring absolute values of V_t_ but merely comparing the relative volumes of distribution between the three tracers. Since each tracer was studied using the same tumor, with only a short time interval between studies, and with CT-defined ROIs, it is likely that the partial volume effects would be similar for all tracers. Finally, SUV was normalized using liver (with moderate-high SSTR expression) and muscle (with low SSTR expression), whereas it would also be advisable to have used brain tissue, as the tumor/brain ratio could be more informative about uptake differences with this tumor type in practice. In any case, with the current normalized values and the result of the kinetic analysis, it can be assumed that at least ^68^Ga-DOTATATE shows increased uptake with regards to the other two tracers, assuming that the tumor/muscle ratio would be considered similar to that of the tumor/brain ratio.

In conclusion, this study demonstrates the ability of the three radiolabeled somatostatin analogues tested to image a human meningioma cell line. Although the V_t_ was relatively similar for ^68^Ga-DOTATATE and ^68^Ga-DOTATOC, ^68^Ga-DOTATATE showed a higher tumor to muscle ratio uptake than the other radiotracers, suggesting that it should be the preferred option for detecting meningiomas.
